# NT-proBNP Predicts Cardiovascular Death in the General Population Independent of Left Ventricular Mass and Function: Insights from a Large Population-Based Study with Long-Term Follow-Up

**DOI:** 10.1371/journal.pone.0164060

**Published:** 2016-10-06

**Authors:** Alexander Dietl, Klaus Stark, Martina E. Zimmermann, Christa Meisinger, Heribert Schunkert, Christoph Birner, Lars S. Maier, Annette Peters, Iris M. Heid, Andreas Luchner

**Affiliations:** 1 Department of Genetic Epidemiology, University of Regensburg, Regensburg, Germany; 2 Department of Internal Medicine II, University Hospital Regensburg, Regensburg, Germany; 3 Institute of Epidemiology II, Helmholtz Zentrum Muenchen, German Research Centre for Environmental Health, Neuherberg, Germany; 4 Deutsches Herzzentrum Muenchen, Technische Universitaet Muenchen, and DZHK (German Centre for Cardiovascular Research), partner site Munich Heart Alliance, Munich, Germany; 5 Klinikum Amberg, Amberg, Germany; Ospedale del Cuore G Pasquinucci Fondazione Toscana Gabriele Monasterio di Massa, ITALY

## Abstract

**Aims:**

B-type natriuretic peptide (BNP) and N-terminal proBNP (NT-proBNP) predict cardiovascular endpoints in patients and all-cause death in the general population. This was assigned to their association with clinical cardiac remodelling defined as changes in size, shape and function of the heart. The aim of this study was to evaluate whether NT-proBNP and BNP were associated with cardiovascular and overall death independent of clinical cardiac remodelling measured by echocardiography as left ventricular hypertrophy (LVH), diastolic dysfunction and left ventricular ejection fraction (EF).

**Methods and Results:**

In a general population-based cohort study from Germany (KORA-S3) with subjects’ baseline age ranging from 25 to 74 years, cardiac morphology and function were assessed as left ventricular mass (LVM), diastolic dysfunction and EF by echocardiography and circulating NT-proBNP and BNP were measured at baseline. In 1,223 subjects with mortality follow-up information, we examined the association of baseline NT-proBNP and BNP with cardiovascular mortality (number of deaths = 52, median follow-up time = 12.9years) using Cox regression without and with adjustment for cardiovascular risk factors, LVM, diastolic dysfunction and EF. The risk of cardiovascular mortality increased with higher NT-proBNP levels measured at baseline (hazard ratio HR = 1.67 per unit increment in logNT-proBNP, p = 2.78*10^−4^, adjusted for age and sex). This increased risk persisted after adjustment for cardiovascular risk factors, LVM, diastolic dysfunction and EF (HR = 1.73; p = 0.047). When excluding subjects with relevant LVH (LVM to body surface area > 149g/m^2^ in men / 122g/m^2^ in women), the NT-proBNP association with mortality was still significant (n = 1,138; number of deaths = 35; HR = 1.48; p = 0.04). We found similar results for BNP.

**Conclusion:**

Our data confirms NT-proBNP and BNP as predictor of cardiovascular mortality in a large general population-based study with long-term follow-up. Our study extends previously published population-based studies to younger and potentially healthier individuals without relevant LVH, diastolic dysfunction or LVD.

## Introduction

B-type natriuretic peptide (BNP) and its equimolarly secreted, more stable N-terminal cleavage product NT-proBNP are increasingly accepted as biomarkers for heart failure: High levels are used for diagnosis of heart failure in emergency units and decreasing levels may indicate treatment success in heart failure patients [1]. Also for the general population, BNP and NT-proBNP are known to reflect cardiac morphology and function as assessed by left-ventricular mass (LVM) and left-ventricular ejection fraction (EF)[2].

Plasma NT-proBNP and BNP levels have already been shown to predict mortality in the general population. In studies like the Framingham Offspring collective [3], the Olmsted County survey[4] and the PREVEND study [5], higher levels of BNP and NT-proBNP at baseline were associated with early all-cause mortality and cardiovascular events in a median follow-up of 5.2 to 7.5 years. These findings have given rise to a lively debate: Is BNP and NT-proBNP predicting mortality solely due to the fact that BNP and NT-proBNP monitor preclinical cardiac processes [6] or are these natriuretic peptides additionally marker of extra-cardiac diseases, as proposed by several recent studies [7–9]. It remains an unsettled issue whether blood NT-proBNP levels solely reflect cardiac processes or also have a role independent of cardiac remodelling.

In the case of any direct association of BNP on mortality independent of cardiac remodelling, at least a part of the association of BNP (or NT-proBNP) with mortality should be independent of measures of cardiac remodelling. In published literature, cardiac remodelling processes [10,11] are complex, interrelated and chamber-specific at the molecular and cellular level. These complex processes manifest clinically. As stated by the International Forum on Cardiac Remodeling [12] and further on commonly accepted [13] and implemented in current guidelines [14,15], clinical cardiac remodelling is precisely defined as changes in size, shape and function of the heart. Common effective echocardiographic measures of such processes are LVM, signs of diastolic dysfunction and EF [12,14,16]. Therefore, a direct role of BNP on mortality independent of cardiac morphology and function can be supported by showing that the association of NT-proBNP levels with mortality persists after adjusting for LVM, diastolic dysfunction and EF in general-population studies. However, only two epidemiological studies with measurements of BNP or NT-proBNP and echocardiography at baseline are currently available reporting follow-up periods of less than six years[3,4]. The aim of our current investigation was to evaluate whether NT-proBNP and BNP were associated with long-term cardiovascular or overall death independent of clinical cardiac remodelling measured as left ventricular hypertrophy (LVH), diastolic dysfunction and left ventricular ejection fraction (EF). We report on our data from the longitudinal general population-based KORA-S3 study (Cooperative Health Research in the Region of Augsburg, Germany), which thoroughly assessed cardiac morphology and function by echocardiography at baseline and provides a unique long-term follow-up of up to 13 years.

## Methods

### Study sample

The echocardiographic study of the German population-based KORA platform (Cooperative Health Research in the Region of Augsburg) was recruited from the third survey (S3) conducted 1994/95. Its design has been described in detail previously [17–20]. Briefly, the study included 1,675 subjects (response 75%) [21] aged 25 to 74 years at baseline based on recruitment from local registries in and around the middle-size cite of Augsburg, Bavaria. Inclusion criteria were a German passport as well as the ability and willingness to provide informed consent. For this investigation, we excluded 452 attendees (unavailable baseline NT-proBNP values, 243 subjects; missing follow-up data, 2 subjects; insufficient M-mode tracings at baseline, 207 subjects) yielding 1,223 for this analysis.

### Ethics statement

The Ethics Committee of the Bavarian Medical Association (Bayerische Landesärztekammer) and the Bavarian commissioner for data protection and privacy (Bayerischer Datenschutzbeauftragter) approved the study. All study participants provide written consent after being informed about the study. All subjects have the option to restrict their consent to specific procedures, e. g. by denying storage of biosamples.

### General data assessment

Socio-demographic status was examined in a standardized face-to-face interview. The presence of arterial hypertension was defined in line with the recently published scientific statement [22] of the American Heart Association as resting systolic blood pressure > 140mmHg and/or diastolic blood pressure > 90mmHg and/or current antihypertensive drug therapy. Diabetes mellitus was established as intake of oral antihyperglycaemic agents or insulin and/or a glycated haemoglobin (HbA_1c_) level equal or above 6.5% as recommended by the American Diabetes Association[23].

### Assessment of cardiac morphology and function by echocardiography

A standardized transthoracic echocardiography was performed in strict accordance with the recommendations of the American Society of Echocardiography (ASE) [24].

LVM was calculated by Devereux formula and analysed in relation to the individual body surface area as approximated by DuBois’ formula [25] (LVMi). For probing purposes, two cut-points for LV hypertrophy were explored. Any left ventricular hypertrophy (LVH) was defined as left ventricular mass to body surface area > 115g/m^2^ for men or > 95g/m^2^ for women, respectively. The cut-off for relevant left ventricular hypertrophy was set to LVMi > 149g/m^2^ (men) / 122g/m^2^ (women) according to the current American and European guidelines [26].

Left atrial (LA) volume was determined according to current guidelines [26] as published elsewhere [27]. The end-systole, blood-tissue interface of the left atrium was traced on the apical four-chamber view. The area under the mitral valve and the inflow of the pulmonary veins were excluded. The volume was calculated using the method of discs (the Simpsons rule)[26]. LA volume was indexed to body surface area (LAVi). LAVi > 34ml/m^2^ defined left atrial enlargement. Peak E–and A-wave velocities of the transmitral flow profile were determined with a PW Doppler sample volume between the tips of the mitral valve leaflets in the apical four-chamber-view. Mitral E/A ratio < 0.75 determined early diastolic dysfunction[28].

Since measurements of LV function by mono- or biplane volumetry (Simpson) were not available in all individuals, the Teichholz formula [29] was used for quantification of left ventricular ejection fraction (EF). EF < 55% was considered as systolic left ventricular dysfunction (LVD) [30].

### Cardiovascular death

The outcome analysed in this study was mortality from any cardiovascular disease (CVD, ICD-9: 390–459). Deaths of study subjects recruited at baseline in the years 1994/95 were ascertained by regularly checking the vital status through population registries inside and outside the study area until December 31rd, 2007. Death certificates were obtained from local health departments and coded for the underlying cause of death by a single trained person using the 9^th^ revision of the International Classification of Diseases (ICD-9). In the analysis, events were censored, if a subject died from a non-cardiovascular disease, was lost to follow-up, or was alive at the end of the follow-up.

### Natriuretic peptide measurements

After venipuncture, all samples were immediately centrifuged, transferred to Eppendorf-cups on ice and frozen at -80° Celsius. No freezing or thawing cycles were performed until final measurement. Measurements were taken from EDTA-plasma samples. BNP measurements have been performed in 2001/2002 and NT-proBNP measurements in 2006. BNP and NT-proBNP were assessed in all samples. Concentrations of plasma NT-proBNP were measured by an electrochemiluminescence immunoassay (elecsys-proBNP, Roche Diagnostics, Risch, Switzerland). Plasma BNP was determined by standard immunoradiometric assay (Shionogi, Osaka, Japan). To convert the unit of pg/ml to ng/l, multiply by 1.

### Statistical methods

The current guidelines of the European Society of Cardiology for the diagnosis of chronic heart failure recommend an exclusion cut-off point of plasma NT-proBNP of 125pg/ml for non-acute patients suspected for heart failure[31]. To further assess the prognostic value of this cut-off point, plasma NT-proBNP levels were dichotomized according to this threshold. To determine the best cut-off value of blood NT-proBNP levels to predict CVD death in our study sample, the receiver operating characteristic (ROC) was computed. Additionally, plasma NT-proBNP and BNP levels were analysed as continuous variables after natural logarithmic transformation (logNT-proBNP, logBNP).

Cox multivariable proportional hazards models were performed to evaluate survival.

With CVD death being our main endpoint, we adjusted the multivariable regression analysis for major cardiovascular risk factors in line with recent publications [3,4] and current guidelines of the American Heart Association [32]. Three regression models were employed: Model I included age and sex as covariates; model II additionally controlled for clinical risk factors (serum creatinine, hypertension, type 2 diabetes, body mass index, BMI, ratio of total to high density lipoprotein); model III extended model II by adding LVMi, LA enlargement, early diastolic dysfunction and EF.

Statistical significance was assigned at a two-sided p-value of less than 0.05. All analyses were carried out with SPSS statistics version 22 (IBM, Armonk, USA).

## Results

### Baseline characteristics

The analysed sample comprised of 1,223 participants with age, sex, baseline measurements of NT-proBNP, baseline assessment of LVM and EF available. Participants’ baseline characteristics are shown in Table 1. Mean age was 49.7±13.7 years and 51.3% of the participants were women. Relevant LVH was detected in 43 (3.5%), and systolic LVD in 124 participants (10.1%).

**Table 1:** Baseline characteristics of the study sample.

Characteristics	Men (n = 596)	Women (n = 627)
Age [years]	49.8±13.9	49.6±13.5
Body-mass index [kg/m^2^]	26.8±3.2	26.4±4.6
Hypertension [%]	45.8	37.2
Diabetes [%]	4.6	3.6
Serum creatinine [mg/dl]	0.84±0.16	0.67±0.14
Total cholesterol [mg/dl]	234.2±43.6	231.3±44.2
High-density cholesterol [mg/dl]	48.0±14.0	60.0±16.6
Left atrial enlargement [%]	6.7	5.4
LVMi [g/m^2^]	96.3±22.3	81.8±19.9
E/A below 0.75 [%]	32.7	36.2
EF [%]	63.6±0.1	65.3±0.1
NT-proBNP [pg/ml] median (IQR)	38.1 (51.1)	66.9 (72.5)
BNP [pg/ml] median (IQR)	5.73 (9.76)	10.16 (11.14)

Shown are mean and standard deviation or proportions (if not indicated otherwise) for the 1,223 analysed subjects separately for men and women.

Left atrial enlargement: left atrial volume index > 34ml/m^2^. LVMi: ratio of left ventricular mass to body surface area. E/A: ratio of mitral valve E wave velocity divided by A wave velocity. EF: left ventricular ejection fraction

### NT-proBNP as predictor for cardiovascular mortality

**Fig 1:**
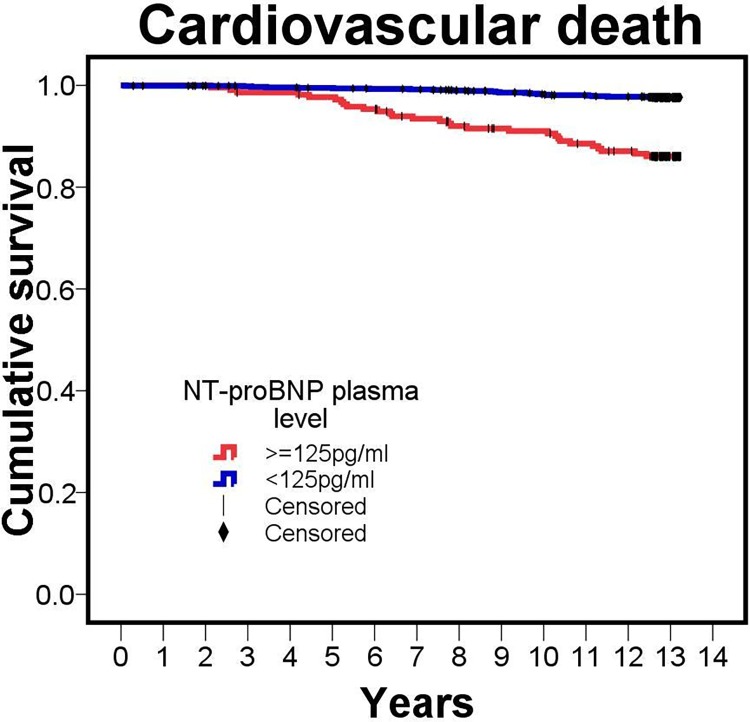
Cumulative survival to cardiovascular death. Kaplan-Meier curve for unadjusted cumulative survival to cardiovascular death according to plasma NT-proBNP dichotomized by the exclusion cut-off point of 125pg/ml recommended by the current guidelines of the European Society of Cardiology for the diagnosis of chronic heart failure [31].

**Fig 2:**
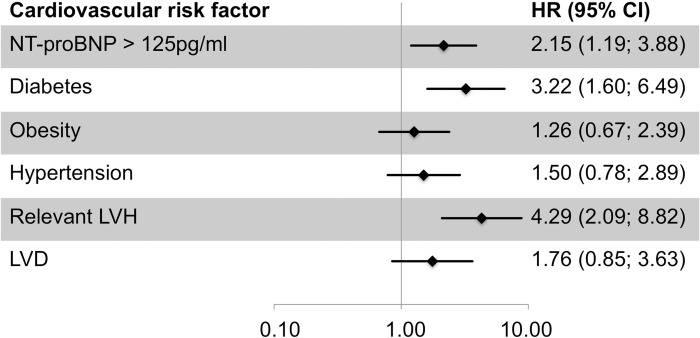
Risk prediction for cardiovascular death by plasma NT-proBNP compared to common cardiovascular risk factors in the general population. Shown are hazard ratio estimates for cardiovascular death adjusted for age and sex. NT-proBNP is dichotomized by the exclusion cut-off point of 125pg/ml recommended by the current guidelines of the European Society of Cardiology for the diagnosis of chronic heart failure [31]. Obesity: body-mass index > 30kg/m^2^. Relevant LVH: left ventricular mass to body surface area > 149g/m^2^ (men) / 122g/m^2^ (women). LVD: systolic left ventricular dysfunction defined as ejection fraction below 55%.

First, we were interested whether our data could confirm the association of NT-proBNP with CVD death. In a median follow-up time of 12.9 years (interquartile range, IQR, 0.4 years, minimum 0.1 year, maximum 13.2 years), 52 of the 1,223 participants died from CVD. For these 52 persons, baseline NT-proBNP was significantly elevated compared to the surviving group (median = 140.8 pg/ml, IQR = 351.0 pg/ml versus median = 51.3 pg/ml, IQR = 64.7 pg/ml, p for difference = 7.7*10^−30^). The mortality according to the commonly used cut-point of 125pg/ml is depicted in Fig 1. A ROC analyses yielded a best cut-off value of blood NT-proBNP concentration to predict CVD death of 114.2pg/ml (sensitivity 60%, specifity 82%). In a Cox regression model, the risk of CVD death was significantly increased by 67% per unit increase in logNT-proBNP when adjusting for age and sex (model I, hazard ratio HR = 1.67, 95% confidence interval, CI = [1.27, 2.21], p = 2.78*10^−4^, Table 2). To derive the NT-proBNP effect on CVD death independent of other cardiovascular risk factors, we further adjusted for serum creatinine, hypertension, diabetes, BMI, and the ratio of total to high density lipoprotein (model II) and found a similar effect (HR = 1.75, 95% CI = [1.33, 2.29], p = 5.17*10^−5^). A comparison of NT-proBNP with traditional cardiovascular risk factors is depicted in Fig 2.

**Table 2:** Increased relative risk for cardiovascular death by higher NT-proBNP levels.

	Hazard Ratio	95% CI	p-Value
Unadjusted	2.26	2.18 to 3.49	2.24*10^−17^
Model I	1.67	1.27 to 2.21	2.78*10^−4^
Model II	1.75	1.33 to 2.29	5.17*10^−5^
Model III	1.73	1.01 to 2.97	0.047

Shown are hazard ratio estimates for cardiovascular death per unit log NT-proBNP and P-values using the Cox proportional hazard regression model without and with various adjustments. Analysed are 1,223 subjects with NT-proBNP measured at baseline including 52 deaths during a long-term follow-up (median 12.9 years, min = 0.1year, max = 13.2years) in a general population 25 to 74 years of age.

Model I: Adjustment for age and sex.

Model II (Clinical risk factors): Adjustment for age, sex, serum creatinine, hypertension, diabetes, BMI and the ratio of total to high-density lipoprotein.

Model III (Clinical and echocardiographic risk factors): As Model II additionally adjusted for LVMi, EF and signs of left ventricular diastolic dysfunction (left atrial enlargement, E/A ratio below 0.75).

### NT-proBNP as predictor for mortality independent of cardiac morphology and function

Second, we wanted to contribute to the debate on the explanation of the NT-proBNP association with cardiovascular mortality. One explanation of this association is that NT-proBNP detects subclinical cardiac adverse processes. Such processes often involve cardiac remodelling and cardiac remodelling can be depicted by measuring LVM, signs of diastolic dysfunction and EF. Therefore, we were interested whether our association of NT-proBNP with CVD death was independent of LVM, LA enlargement, impaired E/A ratio and EF. We found that further adjustment for LVMi, LA enlargement, E/A<0.75 and EF did not erase the significantly elevated risk for CVD death by logNT-proBNP (model III, HR = 1.73, 95% CI [1.01, 2.97], p = 0.047). Our data thus confirms a strong association of NT-proBNP and CVD death in a general population study, which is independent of traditional cardiovascular risk factors and traditional echocardiographic measurements for cardiac remodelling.

### NT-proBNP and cardiovascular mortality in subgroups

Third, we investigated the observed association further in order to see whether more could be learned regarding the potential underlying mechanism. While the Cox model adjusting for covariates already removed the effects of the known cardiovascular risk factors and measures of clinical cardiac remodelling, residual confounding can still be an issue. We thus complemented the previous analyses by subgroup analysis restricting to subjects free of diseases, but also conducted analyses separately for men and women and younger versus older adults.

Similar hazard ratio estimates were found for men and women **(Table 3)**. For female subjects, the association of baseline NT-proBNP blood levels and CVD mortality lost significance which might be explained by few events (number of deaths = 13).

**Table 3:** Increased relative risk for cardiovascular mortality by log NT-proBNP even in the absence of relevant LVH or LVD.

		N	CVD death	HR	95% CI	p-value
Age	≥ 70 years	103	25	2.08	1.38; 3.15	4.80*10^−4^
	< 70 years	1,120	27	1.40	0.92; 2.11	0.114
Sex	Men	596	39	1.68	1.24; 2.28	8.73*10^−4^
	Women	627	13	1.71	0.86; 3.39	0.125
Diabetes	Present	49	10	1.56	0.72; 3.36	0.266
	None	1,149	41	1.70	1.24; 2.31	8.69*10^−4^
Obesity	Present	219	13	1.53	0.88; 2.65	0.129
	None	998	39	1.69	1.23; 2.32	1.07*10^−3^
Any LVH	Present	218	26	1.53	1.07; 2.20	0.021
	None	1,001	26	1.55	0.94; 2.58	0.087
Relevant LVH	Present	43	10	0.80	0.38; 1.70	0.567
	None	1,176	42	1.68	1.22; 2.30	1.31*10^−3^
LVD	Present	124	9	1.75	0.90; 3.40	0.097
	None	1,097	43	1.48	1.02; 2.14	0.041
Any LVH or LVD	Present	311	31	1.64	1.18; 2.28	3.60*10^−3^
	None	906	21	1.03	0.58; 1.80	0.932
Diabetes or Obesity	Present	451	36	1.63	1.20; 2.24	2.11*10^−3^
or any LVH or LVD	None	754	15	0.95	0.49; 1.87	0.887

The analyses of Table 2 were repeated in subgroups defined by sex or age (adjusting by age or sex, respectively) and the presence or absence of predefined cardiovascular risk factors (adjusted by age and sex) again applying a Cox proportional hazard regression model.

CVD death: death from cardiovascular diseases. HR: Adjusted hazard ratio of cardiovascular death per 1 unit increment in log(NT-proBNP) [ln pg/ml]. Obesity: body mass index > 30kg/m^2^. Any LVH: left ventricular hypertrophy defined as left ventricular mass to body surface area >115g/m^2^ (men) / 95g/m^2^ (women). Relevant LVH: left ventricular mass to body surface area > 149g/m^2^ (men) / 122g/m^2^ (women). LVD: systolic left ventricular dysfunction defined as ejection fraction below 55%.

When separating the analysis for older versus younger, the cut-off was defined by the median baseline age (70years) of the 52 participants who died for CVD during the follow-up period. We found more pronounced hazard ratio estimates among the older.

When restricting the analysis to subjects free of diabetes (n = 1,149, number of deaths = 41), NT-proBNP remained a significant and equally strong predictor of cardiovascular death (HR = 1.70, 95% CI = [1.24, 2.31], p = 8.69*10^−4^, Table 3). Quite similar results were found in the subgroup (n = 998) free of obesity (HR = 1.69, 95%CI [1.23, 2.32], p = 1.07*10^−3^, Table 3). This supports the hypothesis that NT-proBNP’s role for death is not primarily due to subclinical diabetes- or obesity-induced processes.

Next, we defined a subgroup free of any LVH (LVMi < 115g/m^2^ in men; LVMi <95g/m^2^ in women, n = 1,001, number of deaths = 26). In this subgroup, the risk for CVD death per unit increase in logNT-proBNP (HR = 1.55) was similar to the risk increase in the entire study sample (HR = 1.67) albeit lower and now missing statistical significance (p = 0.087). The loss of significance might be due to a loss of power with only few deaths remaining in this subgroup. In a less rigorously defined subgroup of “cardiac healthy” subjects just excluding relevant LVH (LVMi < 149g/m^2^ in men; LVMi < 122g/m^2^ in women, n = 1,176; number of deaths = 42), NT-proBNP persisted as strong and significant predictor of CVD death with similar hazard ratio estimate (HR = 1.68, 95%CI = [1.22, 2.30], p = 1.31*10^−3^).

When excluding subjects with LVD (EF < 55%, n = 1,097, number of deaths = 43), the association of baseline NT-proBNP with death remained significant, but the hazard ratio was slightly attenuated (HR = 1.48, 95%CI [1.02, 2.14], p = 0.041).

In summary, effect estimates were very similar across all subgroup analyses with hazard ratios between 1.48 and 1.70. Thus, it is unlikely that the association of NT-proBNP with death was driven purely by either diabetes, obesity, LVH or LVD.

### Plasma NT-proBNP and cardiovascular mortality in combined subgroups

Finally, we wanted to evaluate whether an analysis restricting to a very healthy subgroup still exhibits the NT-proBNP association with CVD death. When excluding participants with any LVH and LVD, NT-proBNP was not associated with CVD death and the effect was nearly abolished (906 individuals, 21 CVD deaths, HR 1.03, P = 0.932). When additionally excluding subjects with diabetes or obesity, we found similar results (754 subjects, 15 deaths, HR = 0.95; p = 0.887). In order to understand these findings, we need to take the distribution of subjects and the distribution of events (= CVD deaths) across the logNT-proBNP values into account (Table A in S1 File). We see that few deaths occur in the very restricted subgroups defined by the absence of diabetes, obesity, LVH and LVD. Additionally, logNT-proBNP values of 91.8% of this very healthy group do not exceed 4.99 ln[pg/ml]. Thus, the lack of association in these subgroup analyses can be due to a loss in power from sample size reduction and a reduction in the variability of logNT-proBNP values in this subgroup (Table A in S1 File).

### Sensitivity analyses using circulating BNP or all-cause mortality

As NT-proBNP and BNP values are highly correlated (Spearman coefficient r = 0.786; p = 1.22*10^−256^), but NT-proBNP levels are better measurable particularly for lower levels (18.9% of BNP values below the lower detection limit in our study), we–as most authors–have analysed NT-proBNP levels, despite the fact that BNP is the biologically active substance. Nevertheless, we have repeated all analyses using BNP values and found a similar association of increased BNP with CVD death (see Tables B and C in S1 File).

Furthermore, we have opted to focus on CVD death as outcome, as we are interested in the cardiac mechanisms; other deaths would include a substantial portion of cancer deaths, which have not been a focus here. However, for comparison to other previously published studies using all-cause mortality, we have also conducted a sensitivity analysis including all deaths and found similar results, with attenuated hazard ratios and similar P-values (Table D in S1 File).

## Discussion

Our data confirms NT-proBNP as strong predictor of cardiovascular mortality independent of LVM, diastolic dysfunction and LVD in a large population-based study with long-term follow-up. This supports the hypothesis that NT-proBNP predicts mortality even beyond traditional measures of clinical LV remodelling and damage.

### Previously published studies on NT-proBNP and risk of death

While there is a bulk of work on NT-proBNP in patients, there are only few studies on natriuretic peptides as predictor of mortality in the general population (Table 4). Wang et al. (2004, Framingham) [3] has set the stage by showing that BNP predicts all-cause mortality in the community for the first time, and that this link persisted even with adjusting for basic measures of cardiac remodelling. A lot of important and substantial work has been done by others to further strengthen Wang’s hypothesis. Not all of these confirmatory analyses have strictly population-based designs or they have targeted composite endpoints: the so far largest study on this issue, the PREVEND study [5], had enriched for study subjects with microalbuminuria, the KIHD study [33] as well as the recently published CaPS study[34] focused on men and the Copenhagen study on the elderly above 55 years [35], albeit without availability of echocardiographic data.

**Table 4:** Published population-based studies investigating blood levels of natriuretic peptides as risk predictors for mortality.

Author	Study	Year of publication	Number of subjects	Age (mean ±SD) [years]	Median follow-up [years]	Natriuretic peptide used (additional measured peptide)	Outcome	Number of events	HR**	P-value	Echo data
Our study	KORA-S3		1,223	49.7±13.7	12.9	NT-proBNP (BNP)	All-cause mortality	99	1.43	0.001	LVM, EF, LVD, LAEn, diastolic function
							CVD death	55	1.67, 1.73†	<0.001,0.047†	
Patterson et al.	CaPS	2015	1,773 **men**	*	15.4	BNP	CVD death	355	1.54‡	<0.001‡	None
Linssen et al.	PREVEND	2010	8,383	49±12.7	7.5	NT-proBNP	All-cause mortality	437	1.22	<0.001	None
Laukkanen et al.	KIHD	2006	905 **men**	55.8±6.6	10	NT-proBNP (NT-proANP)	All-cause mortality	110	1.26	<0.001	None
							CVD death	58	1.41	<0.001	
McKie et al.	Olmsted County	2006	1,991	62±10	5.6	NT-proBNP (BNP)	All-cause mortality	106	1.75, 1.44§	<0.001, 0.014§	LVM, LVD, diastolic/valvular function, LAEn, RWMA
Kistorp et al.	Copenhagen	2005	626	67.9±10.6	5	NT-proBNP	All-cause mortality	94	1.55	0.001	None
Wang et al.	Framingham Offspring	2004	3,346	59±10 (men)/ 58±10 (women)	5.2	BNP (NT-proANP)	All-cause mortality	119	1.27ǁ	0.009ǁ, 0.29¶	LVM, LVD, LAEn

HR: hazard ratio per 1 unit increase in lnNT-proBNP (our study), per third of BNP distribution (CaPS), per 1 unit increase in log_2_NT-proBNP (PREVEND), per 1SD-increment in log variable (KIHD, Olmsted, Copenhagen, Framingham). CVD: cardiovascular disease. LVM: left ventricular mass. EF: left ventricular ejection fraction. LVD: left ventricular systolic dysfunction. LAEn: left atrial enlargement. RWMA: regional wall motion abnormalities.

* predominantly aged 55–69 at baseline.

** adjusted for age and sex (if not indicated otherwise).

† Adjustment for clinical risk factors and echo, in detail: age, sex, serum creatinine, ratio of total to high density lipoprotein, hypertension, diabetes, BMI, LVMi, diastolic dysfunction, EF.

‡ adjusted for age, smoking, diabetes, systolic blood pressure, total cholesterol, total triglycerides, BMI, history of CVD, positive family history of coronary heart disease.

§ Adjustment for clinical risk factors and echo, in detail: age, sex, serum creatinine, total cholesterol, hypertension, diabetes, coronary artery disease, presence of EF <50%, diastolic dysfunction, valvular dysfunction, LVH, LAEn, RWMA.

ǁ adjusted for age, sex, serum creatinine, ratio of total to high-density lipoprotein, hypertension, diabetes, BMI, smoking status.

¶ extends the adjustment of ǁ by adding LVH, LVD, LAEn.

Only two population-based studies–additional to ours—were able to evaluate echocardiographic data and to estimate the mortality prediction by natriuretic peptides without and with adjusting for cardiac remodelling (using a model adjusting for LVM and EF): the Framingham Offspring study[3] by Wang and colleagues, already mentioned above, and the Rochester Epidemiology Project Olmsted County cohort[4]. In these two studies, a random sample of the general population was surveyed with natriuretic peptide and LVM and EF measured at baseline and followed to monitor vital status. This is a similar cohort study design as our KORA-S3 heart failure study.

Our study has a comparable number of all-cause deaths and extends these previous studies by several further insights. Our subjects are on average about 10 years younger and cover a wider age-range of adult subjects. Thus, our confirmatory results are obtained in a younger and potentially healthier study population. In addition, we may report the to date longest follow-up period for this research question (KORA: median of 12.9 years; Framingham: 5.2 years[3]; Olmsted: 5.6 years[4]). We can particularly demonstrate a clear prognostic separation between “normal” and “above-normal” NT-proBNP (>125pg/ml) in the general population. The survival curves do not start to separate until an observation period of > 5 years. Our data indicate that NT-proBNP levels may predict cardiovascular mortality in a general adult population more than a decade before the event. This makes NT-proBNP one of the most powerful predictors for cardiovascular mortality that are easily obtainable even in general practice without echocardiography. Additionally, we focused on cardiovascular death instead of including all-cause mortality, as these are the deaths that we think are those of interest for a direct role of NT-proBNP as supported by Linssen et al.[5] and our own sensitivity analysis. This makes our study the first study in a general adult population being able to depict the prediction of NT-proBNP with specifically CVD death rather than all-cause mortality.

Finally, the mitral E/A ratio was assessed by echocardiography. Thus, our data can show that the association of NT-proBNP and CVD death in the general adult population is even independent of very early signs of diastolic dysfunction.

### On the debate of potential explanations why NT-proBNP predicts mortality

It remains unclear why NT-proBNP is a predictor of all-cause mortality in the apparently healthy, general population. In the PREVEND study, Linssen et al. found that the association was predominantly with cardiovascular events and less with death of other causes, and thus speculated that elevated NT-proBNP levels reflect silent myocardial diseases in the general population[5], which might be conclusive for a cardiac hormone.

The initial hypothesis of the detection of inapparent cardiac disease as reason for the predictive value of natriuretic peptides has been recently doubted by several further data. Natriuretic peptide levels and the development of cardiovascular diseases seem to be more complex and interrelated than expected: experimental research showed BNP as cardiac hormone involved in the development of several extra-cardiac diseases. For example, it directly affects vasculature and metabolism [36,37]. Congruently, very recent epidemiologic studies have reported associations between natriuretic peptides and vascular diseases: in 2015, results from the Heinz-Nixdorf-Recall-study[7] and the Rotterdam study[8] ascertained a predictive value of NT-proBNP for stroke and myocardial infarction. Bower et al. showed that NT-proBNP levels were associated with increased risk of hypertension in the ARIC cohort[38]. Also in 2015, new data from ARIC revealed a strong association of NT-proBNP and abdominal aortic aneurysm as consequence of atherosclerosis[39]. These considerable studies provided a substantial epidemiologic link from natriuretic peptides to extramyocardial diseases, albeit all of these published studies did unfortunately not control for cardiac processes by echocardiography. The Dallas Heart Study [40] is one of two very interesting exceptions: a significant association was reported between NT-proBNP levels and coronary artery calcium scores as marker of coronary artery disease obtained by electron beam computed tomography. This association persisted after adjustment for LVM and EF. In 2016, Pastormerlo and colleagues [9] published a case-control study comparing asymptomatic subjects with hypertension and normotensive controls, where they elaborated a significant association of NT-proBNP levels and carotid intima-media thickness as well as coronary artery calcium score. By echocardiography they determined normal mean values of EF, LVMi and diastolic dysfunction. Thus, these studies reinforced NT-proBNP as potential biomarker of preclinical atherosclerosis which could explain the association of natriuretic peptides with CVD death independent of cardiac remodelling.

LVM, diastolic dysfunction and EF are considered as surrogate parameters of cardiac remodelling–irrespective of whether this cardiac remodelling processes are already associated with clinical symptoms or not. Thus, if there is a direct link between natriuretic peptides and CVD leading up to cardiac remodelling or even bypassing cardiac remodelling, the association of NT-proBNP and CVD death should be independent of LVH, diastolic dysfunction and LVD. Indeed, we find an association of NT-proBNP and BNP with CVD death adjusting for clinical cardiovascular risk factors, LVM, signs of diastolic dysfunction and LVD. We can also report fairly stable risk estimates when omitting one disease or condition at a time. A limitation of our work may be the fact that echocardiographic measurements may lack precision in some uses[18] or the fact that unrecognised confounders had an effect[41]. However, removing those with relevant LVH at baseline did not change the risk estimate; removing more of those with either normal LVH or those with left ventricular dysfunction decreased the risk estimate slightly. These results strengthen natriuretic peptides as risk predictor of cardiovascular death. They may integrate risks derived from several cardiovascular risk states which might escape traditional echo quantification.

### Strengths and limitations

A strength of our study is the population-based approach, the highly standardized procedures, conduct by experienced, trained and quality controlled staff, and the particularly long follow-up of more than a decade. Another strength is that echocardiography was conducted as part of the study protocol, which makes it one of the few population-based studies that can relate events happening a decade later to cardiac measurements at baseline.

We report a prevalence of 10.1% for systolic left ventricular dysfunction which might appear high for the rather young study population. As we intended to correct our analyses even for very early manifestations of systolic LVD, we followed the very strict guidelines of 2005 defining systolic LVD as EF below 55%[30]. To ensure comparability with other definitions, we calculated additionally the prevalence of systolic LVD in our study sample according to cut-offs used by other large population-based samples (Table E in S1 File). The prevalence of systolic LVD in our study is consistently lower than reported by the Rotterdam study[42], the Olmsted County sample [4]and the Strong-Heart Study[43] which is in line with our population being younger and more healthy.

Concerning the echocardiographic measurements our study is subject to the inherent limitations of any of the few studies implying cardiovascular imaging technologies and exhibiting such a follow-up period. Echocardiography was done according to current guidelines and using the up-to-date equipment of 1994/1995. However, the current guidelines of the American Society of Echocardiography and the European Association of Cardiovascular Imaging published in 2015 [26] still recommend the linear method to determine LVM as in 1994/1995 and reinforced this method for every-day screen and explicitly for large population studies as published data did not state a clear advantage of another technique [44]. Hence, the LVM measurement in 1994/1995 should not be a major drawback to define LVH.

Key structural alterations of diastolic dysfunction (LVH and LA enlargement) were assessed according to the current heart failure [31] and echocardiographic guidelines [45]. For the detection of very early diastolic dysfunction without LA enlargement the E/A ratio was used as proposed by Redfield [28] and implemented in current guidelines [16,45]. In our study, tissue Doppler imaging was not conducted, which impedes the grading of diastolic dysfunction (I°-III°). However, adjustment for diastolic dysfunction per se was possible and included in the analyses.

Regarding the measurement of EF, the biplane method of disks is recommended by the guidelines to accurately determine EF. We derived global LV function from linear M-Mode measurements which is less precise in the presence of wall motion abnormalities than the recommended method of disks. Last year’s guidelines concede that the older method may provides useful information in healthy subjects [26]. As only 6 of 1,223 participants in our study reported a history of myocardial infarction, it should be a relatively healthy population with a low prevalence of regional wall motion abnormalities. Thus, the shortcoming of lacking EF determined by biplane method cannot be corrected but should play a minor role in the context of this population based study.

## Conclusion

Our data confirms NT-proBNP and BNP as strong and independent predictors of cardiovascular disease death as well as all-cause mortality during long-term follow-up in a large general population-based study. Our study also indicates that this prediction is largely independent of echocardiographic parameters of relevant cardiac remodelling and already occurs within and slightly above the normal range. Our data strengthens the hypothesis that natriuretic peptides monitor subtle preclinical cardiovascular processes very early on and beyond traditional echocardiographic markers. Given our particularly long follow-up, we were able to depict NT-proBNP as a predictor of death more than a decade before the event, which makes it one of the most powerful predictors that can be easily obtained in general practice.

## Supporting Information

S1 File (DOCX)
