# Supplementary material for: NT-proBNP Predicts Cardiovascular Death in the General Population Independent of Left Ventricular Mass and Function: Insights from a Large Population-Based Study with Long-Term Follow-Up
Source: PLoS One. 2016 Oct 6;11(10):e0164060. doi: 10.1371/journal.pone.0164060 (PMC5053441; doi:10.1371/journal.pone.0164060)
Supplement: S1 File — (DOCX) [file pone.0164060.s001.docx]

**S 1 File Table A: Number of CVD deaths distributed across log NT-proBNP values by subgroups.**

The percentage of deaths during follow-up is shown by subgroup (number of deaths and total number of subjects in each subgroup in parentheses).

| logNT-proBNP | 1 to 1.99 | 2 to 2.99 | 3 to 3.99 | 4 to 4.99 | 5 to 5.99 | 6 to 6.99 | 7 to 7.99 | 8 to 8.99 | All |
| --- | --- | --- | --- | --- | --- | --- | --- | --- | --- |
| NT-proBNP levels | 5.0-7.2 | 7.5-20.0 | 20.1 - 54.6 | 54.7 - 146.8 | 149.6 - 401.6 | 403.5 - 1095 | 1367 - 1763 | 3050 | 5 - 3050 |
| All subgroups | 0% (0/8) | 1.4% (2/147) | 1.7% (8/479) | 4.5% (19/418) | 6.6 % (9/137) | 39.3% (11/28) | 40.0% (2/5) | 100% (1/1) | 4.3% (52/1,223) |
| Women | (0/0) | 0% (0/24) | 0.4% (1/226) | 1.8% (5/271) | 4.3% (4/93) | 25% (3/12) | 0% (0/1) | (0/0) | 2.1% (13/627) |
| Men | 0% (0/8) | 1.6% (2/123) | 2.8% (7/253) | 9.5% (14/147) | 11.4% (5/44) | 50.0% (8/16) | 50% (2/4) | 100% (1/1) | 6.5% (39/596) |
| Age ≥ 70yr | (0/0) | 0% (0/1) | 7.7% (1/13) | 22.9% (8/35) | 15.0% (6/40) | 66.7% (8/12) | 100% (1/1) | 100% (1/1) | 24.3% (25/103) |
| Age < 70yr | 0% (0/8) | 1.4% (2/146) | 1.5% (7/466) | 2.9% (11/383) | 3.1% (3/97) | 18.8% (3/16) | 25% (1/4) | (0/0) | 2.4% (27/1,120) |
| Diabetes | (0/0) | 33.3% (1/3) | 6.7% (1/15) | 15.8% (3/19) | 30% (3/10) | 100% (2/2) | (0/0) | (0/0) | 20.4% (10/49) |
| No Diabetes | 0% (0/8) | 0.7% (1/142) | 1.5% (7/456) | 4.1% (16/392) | 4.9% (6/122) | 34.8% (8/23) | 40% (2/5) | 100% (1/1) | 3.6% (41/1,149) |
| Obesity | (0/0) | 6.3% (1/16) | 2.5% (2/79) | 3.7% (3/81) | 9.4% (3/32) | 44.4% (4/9) | 0% (0/2) | (0/0) | 5.9% (13/219) |
| No Obesity | 0% (0/8) | 0.8% (1/131) | 1.5% (6/399) | 4.8% (16/333) | 5.8% (6/104) | 36.8% (7/19) | 66.7% (2/3) | 100% (1/1) | 3.9% (39/998) |
| Any LVH | (0/0) | 0% (0/4) | 6.1% (3/49) | 9.1% (8/88) | 9.4% (5/53) | 38.9% (7/18) | 40% (2/5) | 100% (1/1) | 11.9% (26/218) |
| No LVH | 0% (0/8) | 1.4% (2/143) | 1.2% (5/429) | 3.4% (11/327) | 4.8% (4/84) | 40% (4/10) | (0/0) | (0/0) | 2.6% (26/1,001) |
| LVD | 0% (0/1) | 0% (0/13) | 0% (0/37) | 4.9% (2/41) | 5.0% (1/20) | 50.0% (4/8) | 33.3% (1/3) | 100% (1/1) | 7.3% (9/124)) |
| No LVD | 0% (0/7) | 1.5% (2/133) | 1.8% (8/442) | 4.5% (17/376) | 6.8% (8/117) | 35.0% (7/20) | 50% (1/2) | (0/0) | 3.9% (43/1,097) |
| Any of the listed diseases | 0% (0/1) | 3.4% (1/29) | 2.8% (4/145) | 7.1% (12/170) | 8.9% (7/79) | 42.9% (9/21) | 40% (2/5) | 100% (1/1) | 8.0% (36/451) |
| None of the listed diseases | 0% (0/7) | 0.9% (1/115) | 1.2% (4/330) | 2.9% (7/240) | 3.6% (2/56) | 16.7% (1/6) | (0/0) | (0/0) | 2.0% (15/754) |

CVD death: death from cardiovascular diseases. Obesity: body mass index > 30kg/m^2^. Any LVH: Left ventricular hypertrophy; left ventricular mass to body surface area > 115g/m^2^ (men) or > 95g/m^2^ (women). LVD: systolic left ventricular dysfunction defined as ejection fraction below 55%. Any / none of the listed diseases: at least one / none of the diseases diabetes, obesity, LVH or LVD.

**S1 File Table B: Increased** **relative risk for cardiovascular death by higher BNP levels.**

The analyses of Table 2 were repeated using blood BNP levels in place of NT-proBNP levels. Shown are hazard ratio estimates for cardiovascular death per unit log BNP and P-values using the Cox proportional hazard regression model without and with various adjustments. Analysed are 1,223 subjects including 52 deaths during a long-term follow-up (median 12.9 years, min = 0.1year, max =13.2years) in a general population 25 to 74 years of age.

|  | Hazard Ratio | 95% CI | p-Value |
| --- | --- | --- | --- |
| Unadjusted | 2.46 | 1.92 to 3.14 | 1.06*10^-12^ |
| Model I | 1.49 | 1.15 to 1.94 | 2.76*10^-3^ |
| Model II | 1.56 | 1.20 to 2.03 | 7.97*10^-4^ |
| Model III | 1.37 | 0.96 to 1.95 | 0.079 |

Model I: Adjustment for age and sex.

Model II (Clinical risk factors): Adjustment for age, sex, serum creatinine, hypertension, diabetes, BMI and the ratio of total to high-density lipoprotein.

Model III (Clinical and echocardiographic risk factors): As Model II additionally adjusted for LVMi, EF and signs of left ventricular diastolic dysfunction (left atrial enlargement, E/A ratio below 0.75).

**S 1 File Table C: Increased relative risk for cardiovascular mortality by log BNP even in the absence of relevant LVH.**

The analyses of Table 2 were repeated using blood BNP levels in place of NT-proBNP in subgroups defined by sex or age (adjusting by age or sex, respectively) and the presence or absence of predefined cardiovascular risk factors (adjusted by age and sex) again applying a Cox proportional hazard regression model.

|  |  | N | CVD death | HR | 95% CI | p-value |
| --- | --- | --- | --- | --- | --- | --- |
| Age | ≥ 70 years | 103 | 25 | 1.81 | 1.22; 2.69 | 3.26*10^-3^ |
|  | < 70 years | 1,120 | 27 | 1.27 | 0.87; 1.84 | 0.218 |
| Sex | Men | 596 | 39 | 1.58 | 1.18; 2.13 | 2.23*10^-3^ |
|  | Women | 627 | 13 | 1.19 | 0.67; 2.12 | 0.551 |
| Diabetes | Present | 49 | 10 | 1.52 | 0.82; 2.81 | 0.183 |
|  | None | 1,149 | 41 | 1.48 | 1.10; 2.01 | 1.08*10^-2^ |
| Obesity | Present | 219 | 13 | 1.79 | 1.07; 3.00 | 2.64*10^-2^ |
|  | None | 998 | 39 | 1.38 | 1.02; 1.88 | 3.58*10^-2^ |
| Any LVH | Present | 218 | 26 | 1.37 | 0.97; 1.95 | 0.077 |
|  | None | 1,001 | 26 | 1.45 | 0.98; 2.15 | 0.066 |
| Relevant LVH | Present | 43 | 10 | 0.83 | 0.43; 1.60 | 0.573 |
|  | None | 1,176 | 42 | 1.49 | 1.11; 2.00 | 8.68*10^-3^ |
| LVD | Present | 124 | 9 | 1.86 | 0.97; 3.55 | 0.061 |
|  | None | 1,097 | 43 | 1.30 | 0.81; 2.09 | 0.275 |
| Any LVH or LVD | Present | 311 | 31 | 1.47 | 1.06; 2.03 | 0.022 |
|  | None | 906 | 21 | 1.21 | 0.78; 1.86 | 0.396 |
| Diabetes or Obesity | Present | 451 | 36 | 1.49 | 1.10; 2.03 | 0.011 |
| or any LVH or LVD | None | 754 | 15 | 1.07 | 0.64; 1.77 | 0.804 |

CVD death: death from cardiovascular diseases. HR: Adjusted hazard ratio of cardiovascular death per unit increment in log(BNP) [ln pg/ml]. Obesity: body mass index > 30kg/m^2^. Any LVH: left ventricular hypertrophy defined as left ventricular mass to body surface area >115g/m^2^ (men) / 95g/m^2^ (women). Relevant LVH: left ventricular mass to body surface area > 149g/m^2^ (men) / 122g/m^2^ (women). LVD: systolic left ventricular dysfunction defined as ejection fraction below 55%.

**S 1 File Table D: Increased** **relative risk for all-cause death by higher NT-proBNP levels.**

We repeated the analyses of Table 2 using all-cause death as outcome variable instead of cardiovascular death. Shown hazard ratio estimates for all-cause death per unit log NT-proBNP. Analysed are 1,223 subjects with NT-proBNP measured at baseline including 99 all-cause deaths during a long-term follow-up (median 12.9 years) in a general population 25 to 74 years of age.

|  | Hazard Ratio | 95% CI | p-Value |
| --- | --- | --- | --- |
| Unadjusted | 2.19 | 1.82 to 2.62 | 2.69*10^-17^ |
| Model I | 1.43 | 1.15 to 1.77 | 1.10*10^-3^ |
| Model II | 1.46 | 1.18 to 1.81 | 5.29*10^-4^ |
| Model III | 1.37 | 0.96 to 1.96 | 0.084 |

Model I: Adjustment for age and sex.

Model II (Clinical risk factors): Adjustment for age, sex, serum creatinine, hypertension, diabetes, BMI and the ratio of total to high-density lipoprotein.

Model III (Clinical and echocardiographic risk factors): As Model II additionally adjusted for LVMi, EF and signs of left ventricular diastolic dysfunction (left atrial enlargement, E/A ratio below 0.75).

.

**S 1 File Table E: Lower prevalence of left ventricular dysfunction in the current study compared to published population-based studies**

| Study | Rotterdam [42] | Our study (KORA S3) | Olmsted County [4] | Our study (KORA S3) | Strong Heart Study [43] | Our study (KORA S3) |
| --- | --- | --- | --- | --- | --- | --- |
| LVD definition | FS ≤ 25 % | | EF < 50% | | EF < 54% | |
| LVD prevalence [%] | 4 | 3 | 5 | 3 | 14 | 8 |

LVD: left ventricular dysfunction. FS: fractional shortening. EF: left ventricular ejection fraction.
